# Methodological quality and clinical applicability of long COVID guidelines and scientific statements: a systematic review using AGREE II and AGREE-REX

**DOI:** 10.3389/fpubh.2026.1930005

**Published:** 2026-09-29

**Authors:** Ricardo Montoya Monsalve, Sofía Campuzano Cortina, Manuela Jiménez Cárdenas

**Affiliations:** 1 Research Group “Psicología, Salud y Sociedad”, School of Social Sciences and Humanities, CES University, Medellín, Colombia; 2 School of Social Sciences and Humanities, CES University, Medellín, Colombia; 3 CES University, Medellín, Colombia

**Keywords:** COVID-19, evidence-based medicine, GRADE approach, post-acute COVID-19 syndrome, practice guidelines as topic, quality of health care, SARS-CoV-2, systematic reviews as topic

## Abstract

**Introduction:**

Long COVID is projected to affect 0.6 to 1.0% of the population between 2025 and 2030. This study reviewed the methodological quality, clinical applicability and content of the care recommendations issued for adults ≥18 years with long COVID in clinical practice guidelines (CPGs) and scientific statements (SSs).

**Methods:**

Registered in PROSPERO (CRD420261324906). PubMed, Scopus, trip, five guideline repositories and national health portals were searched between February and April 2026, and PubMed and Scopus were updated on 2 September 2026. Eligible documents were CPGs and SSs on adults with suspected or confirmed long COVID, published in English or Spanish. Screening and full-text review were duplicated independently; data were extracted by one reviewer and verified by a second. Methodological quality was appraised with AGREE II, reporting the six standardised domain scores separately, each rescaled within its domain and never summed into an overall score; applicability was appraised with AGREE-REX. A narrative synthesis covered five predefined care domains.

**Results:**

Twenty documents were appraised, 19 from the initial search and one from the update. From the 19 initial documents, 510 recommendations were extracted across five NICE-derived domains, mostly clinical management (63.3%) and care planning (29.6%). Quality was uneven (rigor of development 16.7–97.9%; applicability 0.0–95.8%; editorial Independence median 100%), and only one document reported a GRADE-compatible evidence-to-decision framework. Four documents were prioritised and contributed 99 recommendations; they scored higher than the remaining corpus in all six domains, yet applicability remained their weakest (68%). AGREE-REX showed recommendations directed far more clearly at clinicians than at patients or decision-makers. Content converged on shared decision-making, multidisciplinary rehabilitation, patient-centered care and return-to-work support.

**Discussion:**

Guidance on long COVID is abundant but methodologically uneven, and the unevenness is concentrated in how evidence is linked to recommendations and in applicability. The corpus offers well-formulated clinical content whose evidence-to-decision reasoning and implementation requirements are seldom reported, and it therefore requires formal contextual adaptation, for example through GRADE-ADOLOPMENT, before use. Key limitations are the scarcity of guidance from low- and middle-income countries and the diversity of development methods.

**Systematic review registration:**

https://www.crd.york.ac.uk/prospero/display_record.php?ID=CRD420261324906, Identifier CRD420261324906.

## Background

Despite declining estimates of Long COVID prevalence, the condition remains a major global public health challenge, with substantial consequences for disability and health-related quality of life (1, 2). The Organisation for Economic Co-operation and Development (OECD) estimated that in 2021 approximately 5% of the population in member countries experienced Long COVID and projected that 0.6–1.0% would remain affected between 2025 and 2030, with an associated annual cost of approximately US$11 billion (3). Consistent reductions have also been reported among COVID-19 survivors. For example, Shah (4) reported a decline in prevalence among U.S. adults from 19.7% in 2022 to 13.7% in 2024, alongside an increase in recovery from 51.2 to 59.7% (4).

Evidence syntheses suggest that symptom remission occurs within approximately 1 year for many individuals (5), whereas prolonged courses are more common among patients with comorbidities, frailty, or multiple risk factors. However, data on symptom duration remains limited, and longer follow-up studies are needed to characterise recovery trajectories. The long-term consequences for individuals and health systems are still incompletely understood (5), and the evidence base for treatment continues to evolve (6). Nevertheless, there is broad consensus that people living with Long COVID require comprehensive, and integrated care to address heterogeneous health needs and mitigate health and economic burden, with prioritisation by public health authorities (3).

Since early reports of persistent symptoms after acute SARS-CoV-2 infection, the evidence base informing management has expanded rapidly. For instance, the living evidence synthesis by Atkins et al. (6) identified 218 randomized controlled trials and 56 systematic reviews by June 2024 (6). In response, clinical practice guidelines (CPGs) and scientific statements (SSs) have been developed across diverse settings to guide the care of people living with Long COVID. A comprehensive synthesis of these recommendations could support clinicians and decision-makers, particularly in contexts with limited resources to develop or update guidance and potentially inform “ADOLOPMENT” approaches based on high-quality, contextually relevant CPGs or SSs.

However, despite the prolonged proliferation of CPGs and SSs on COVID-19, the obtainable evidence remains fragmented across multiple documents that are diverse in their institutional origin, clinical scope, and methodological rigor. Up to now, no systematic synthesis that integrates the recommendations from these sources in a structured manner is available, and this scatter makes it difficult to clearly identify specific recommendations, their level of evidence, and under what conditions they can be applicable.

Furthermore, the methodological variability among the existing documents, fluctuating from expert consensus to GRADE-based guidelines, demands not only to compile the available recommendations but also to systematically evaluate their quality and clinical applicability as a requirement to any adolopment process. In this regard, a systematic review of CPGs and SSs that synthesises, classifies, and critically evaluates the available recommendations represents a methodologically relevant and clinically necessary contribution to guiding the comprehensive care of people with Long COVID. Previous reviews have appraised Long COVID guidance: Marshall-Andon et al. (7) combined AGREE II and AGREE-REX in the narrower field of post-COVID rehabilitation, and Luo et al. (8, 9) applied AGREE-based appraisal to international guidance and to recent US, Canadian and Australian documents. The present review differs in the breadth of its scope, which is not restricted to a single clinical area, and in synthesizing guidance at the level of the individual recommendation rather than the document.

Accordingly, this study aimed to systematically review care recommendations for adults ≥18 years with suspected or confirmed Long COVID (new-onset, persistent, or relapsing symptoms that last for at least 3 months after SARS-CoV-2 infection, and cannot be explained by an alternative diagnosis), reported in CPGs and SSs published since 2020 by national or international institutions and professional societies, including, with or without a comparator, interventions related to treatment, psychoeducation, rehabilitation, work or academic reintegration, and/or integrated care; this by evaluating the methodological attributes of the various documents using the AGREE II and AGREE-REX instruments, and characterising the evidence-based recommendations of good practice distributed in the domains of care planning, clinical management, follow-up and discharge, continuity of care and patient information, and organisation of services. Throughout this article the term Long COVID is used as the primary designation; post-COVID-19 condition and post-acute sequelae of SARS-CoV-2 infection (PASC) are treated as synonyms and appear only where a source document uses them.

## Methods

### Protocol and registration

We conducted a systematic review of recommendations reported in CPGs and SSs, following the methodological guidance proposed by Johnston et al. (10). The review protocol was registered in PROSPERO (CRD420261324906). We report this review in accordance with the PRISMA 2020 statement (11).

### Eligibility criteria

We included CPGs and SSs meeting the following PICAR criteria: For this review, a clinical practice guideline was defined as a document reporting an explicit set of recommendations informed by a systematic review of the evidence and a formal assessment of benefits and harms, and a scientific statement as a document reporting synthesised expert opinion issued by an organised group, without a formal evidence-to-recommendation process. Both were eligible, and document type was recorded and considered when interpreting the quality appraisals. *Population*: adults ≥18 years with suspected or confirmed Long COVID (systemic disease state occurring after a SARS-CoV-2 infection that can be new, persistent, or relapsing symptoms that last for at least 3 months and cannot be explained by an alternative diagnosis and appear); *Intervention*: any technique, method, or technology related to treatment, psychoeducation, rehabilitation, return to school/work, or integrated care for people living with Long COVID; *Comparator*: any comparator or no comparator; *Guideline attribute*: CPGs or SSs issued by national or international institutions or professional societies, published from 2020 onwards; published in English or Spanish; *Recommendation*: evidence-based recommendations or good practice statements. To avoid double counting due to overlapping guidance when multiple documents addressed the same recommendation, we prioritised those that were more up to date, had higher methodological quality, and presented lower risk of conflicts of interest, excluding less rigorous or redundant documents from the synthesis. This approach followed Cochrane guidance for managing overlapping evidence (12). A dual-reviewer process was conducted across all stages of screening and selection. Quality appraisal was based on the standardised domain scores of the AGREE II instrument; AGREE-REX was applied after prioritisation and therefore did not contribute to the selection of documents.

In accordance with PRISMA guidelines, the retrieved documents and recommendations were classified and allocated to specific synthesis components based on National Institute for Health and Care Excellence (NICE) clinical criteria. Specifically, the evidence was grouped into five established clinical domains: Care Planning, Management, Monitoring and Follow-up, Information Sharing and Continuity of Care, and Service Organisation. This precise classification enhances the reproducibility of our synthesis by grouping the evidence into clearly defined clinical themes.

### Information sources and search strategy

We conducted a systematic search for CPGs and SSs in PubMed, Scopus and Trip, and across guideline repositories and developer websites, including the Guidelines International Network (GIN), NICE, SIGN, the American College of Physicians (ACP), and the WHO. We also searched official government health portals from Spain, Mexico, Canada, and the United States. We additionally searched the eCOVID-19 Living Map of Recommendations (RecMap^1^), a living repository that compiles COVID-19 and post-COVID-19 recommendations internationally and appraises source guidelines with AGREE II, together with its Canadian post-COVID-19 condition instance.^2^ In PubMed and Scopus, we combined the term “Long COVID” and related synonyms with the broad CPG search filter developed by the Canadian Agency for Drugs and Technologies in Health (CADTH) (13, 14). As additional sources, we performed citation and reference checking of included documents.

The initial search was performed across 2 databases (PubMed on 27 February 2026; Scopus on 5 March 2026) and 5 registers, all searched on 7 April 2026. Consultation of the eCOVID-19 RecMap identified post-COVID guidance from nine issuing organisations not represented in the corpus. Applying the prioritisation criteria to the AGREE II appraisals published by that repository, seven scored below the 70% threshold in Rigor of Development, one was not a guidance document but a qualitative study, and one, a national guideline already represented in the corpus, was a duplicate. One document, the World Health Organisation clinical management guidance, met the threshold and was assessed for eligibility.

Following recommendations received during peer review and given the rapidly evolving guidance landscape for Long COVID, the database searches were re-run on 2 September 2026 in PubMed and Scopus using the corrected search syntax (see Registration and protocol, Deviation 2). After removing duplicates and records already screened in the initial search, 298 new records were screened independently by two reviewers against the same eligibility criteria, with disagreements resolved by consensus (observed agreement 98.7%, Cohen’s kappa 0.50, attenuated by the very low proportion of eligible records). Two records advanced to full-text assessment. Three further records retrieved by the updated search were duplicates of documents already appraised in the initial corpus, reflecting reclassification of their publication type in the source database rather than new guidance. Of the two records assessed at full text, one, the international clinical practice guideline for long COVID prevention and treatment developed by the Multidisciplinary Working Group on Long COVID Practice Guidelines (15), met the eligibility criteria and underwent AGREE II appraisal, independently rated by two reviewers and resolved by consensus. It scored 40.6% in Rigor of Development, below the 70% threshold specified in the prioritisation criteria, and was therefore retained as complementary evidence rather than carried forward to the recommendation synthesis. The updated search therefore increased the appraised corpus from 19 to 20 documents and the complementary-evidence group from 15 to 16 but did not alter the four documents prioritised for the evidence synthesis, the 510 recommendations extracted, or the AGREE II domain score ranges reported for the corpus.

Full search strategies for all sources, including the dates and yields of the updated search, are provided in Supplementary material 1.

### Selection process

Metadata from all identified records were exported to Microsoft Excel for management, and duplicates were removed using key identifiers (e.g., DOI/PMID/title) with manual verification. Two reviewers independently screened titles and abstracts. Agreement between reviewers at the full-text stage was substantial (observed agreement 84.6%, Cohen’s kappa 0.696, *n* = 78 records with paired decisions). Potentially eligible records underwent full-text assessment to determine final inclusion. Disagreements were resolved through discussion and consensus; when required, documents were jointly reviewed to reach a final decision.

### Prioritisation for the evidence synthesis

The registered protocol (PROSPERO CRD420261324906) specified that previous versions of a guideline or scientific statement were not eligible (one departure from this rule was applied and is reported below and among the protocol deviations), and that the evidence synthesis would follow a hierarchical order giving precedence to recommendations issued by clinical practice guidelines of high methodological quality that applied GRADE. That hierarchy was operationalised as a four-step procedure applied sequentially to the appraised corpus, together with a rule governing precedence within the prioritised set.

*Step 1, methodological quality.* Documents scoring below 70% in AGREE II Domain 3 (Rigor of Development) were not carried forward. Restricting the synthesis to guidance meeting a minimum quality threshold is an accepted form of methodological tailoring in systematic reviews of guidelines (10), and the threshold applied here follows published practice.

*Step 2, breadth of scope.* Documents whose thematic scope was restricted to a single clinical manifestation, rather than addressing the comprehensive assessment and management of Long COVID, were not carried forward.

*Step 3, thematic complementarity.* Among the documents passing Steps 1 and 2, priority was given to those contributing recommendations in a clinical domain, or of a type, not already represented in the prioritised set. Complementarity was assessed against the five predefined NICE clinical domains (Care Planning; Management; Follow-up, Monitoring and Discharge; Sharing Information and Continuity of Care; and Service Organisation) and against the type of recommendation contributed, distinguishing evidence-based recommendations from good practice or consensus statements, and clinical from service-organisation recommendations. Documents whose domains and recommendation types were already fully covered by a document of equal or higher methodological quality already prioritised were not carried forward, in order to avoid double counting of overlapping guidance, consistent with Cochrane guidance for managing overlapping evidence (12).

*Step 4, precedence within the prioritised set.* Where prioritised documents addressed the same clinical question, precedence was given to recommendations issued under a GRADE-compatible evidence-to-decision framework. This criterion governed the ordering of recommendations within the synthesis and was not applied as an eligibility filter, since only one of the prioritised documents reported such a framework.

Exceptions to Steps 1 and 2 were permitted where a clinical domain would otherwise have remained uncovered by the prioritised set. Any exception required the agreement of both reviewers, was applied only when no document meeting the criteria addressed the domain concerned and is reported explicitly below and among the protocol deviations. Recommendations from documents that were not prioritised were retained and used as complementary evidence wherever the prioritised documents provided insufficient coverage of a thematic domain.

First, Fine et al. (16), the AAPM&R multidisciplinary collaborative consensus guidance statement on the assessment and treatment of cognitive symptoms in PASC, met neither Step 1 (62.5% in Rigor of Development) nor Step 2 (scope restricted to cognitive symptoms), and was nevertheless prioritised. Cognitive symptoms are among the most frequently reported and functionally disabling manifestations of Long COVID, and no document meeting Steps 1 and 2 provided comparable coverage of their assessment and management, the remaining prioritised documents addressing this domain only through general screening statements. The synthesis also incorporates one document that did not meet the methodological threshold applied in this review: Fine et al. (16) scored 62.5% in Rigor of Development, and its recommendations on cognitive symptoms should therefore be read as expert consensus rather than as evidence-based guidance.

Second, the registered record specified that previous versions of a guideline or scientific statement were not eligible. Kim et al. (17), the preliminary guideline of the Korean Society of Infectious Diseases, was subsequently updated by the same society in 2024 and 2025. Both the preliminary version and the most recent one, Seo et al. (18), were nevertheless appraised, and the preliminary version rather than the update was carried forward to the prioritised set. Seo et al. (18) scored 68.8% in Rigor of Development, below the 70% threshold, and 0.0% in Applicability, the lowest score in the corpus, with all four Domain 5 items at the minimum, consistent with the absence of any reported implementation barriers, application tools, resource implications or monitoring criteria; Kim et al. (17) scored 95.8 and 75.0% in the same two domains. Because applicability is directly relevant to a review intended to inform contextual adaptation, retaining the earlier version preserved recommendations that would otherwise have entered the synthesis without any accompanying implementation guidance. This is a departure from the eligibility rule as registered and not an application of it: under that rule, Kim et al. (17) would have been excluded as a superseded version. The departure is reported among the protocol deviations (Deviation 9). Recommendations from Seo et al. (18) were used as complementary evidence, and both versions are reported in Table 1 so that the effect of this decision can be inspected.

**Table 1:** Characteristics of the appraised guidance documents (*n* = 20).

Title	Year	Issuing body	Country/jurisdiction	Health system type	Method of recommendation development	Population	Context	Conflict of interest	AGREE II D3 Rigor (%)	Prioritised
NICE guideline on long COVID	2024	National Institute for Health and Care Excellence (NICE)	United Kingdom	National health service (tax-funded, universal)	Evidence-based clinical guideline	Adults with Long COVID	Clinical and service organisation	None	85.4	Yes
Preliminary Guidelines for the Clinical Evaluation and Management of Long COVID	2022	Korean Society of Infectious Diseases	Republic of Korea	National health insurance (single payer)	Expert multidisciplinary consensus	Adults with Long COVID	Clinical	None	95.8	Yes
Italian good practice recommendations on management of persons with Long-COVID	2023	Istituto Superiore di Sanità [Giuliano et al. (28)]	Italy	National health service (tax-funded, universal)	Multidisciplinary expert consensus without GRADE	Adults with Long COVID	Clinical and service organisation	None	79.2	Yes
Multi-disciplinary collaborative consensus guidance statement on the assessment and treatment of cognitive symptoms in patients with PASC	2022	AAPM&R Multi-Disciplinary PASC Collaborative [Fine et al. (16)]	United States	Mixed public–private insurance	Multidisciplinary expert consensus without GRADE	Adults with Long COVID and cognitive symptoms	Clinical	Institutional funding disclosed, unrelated to this work	62.5	Yes
ESCMID rapid guidelines for assessment and management of long COVID	2022	European Society of Clinical Microbiology and Infectious Diseases [Yelin et al. (24)]	Europe (multinational)	Multiple systems	Rapid guideline	Adults with Long COVID	Clinical	None	97.9	No
Recommendations for the recognition, diagnosis, and management of long COVID: a Delphi study	2021	Delphi panel [Nurek et al. (25)]	United Kingdom	National health service (tax-funded, universal)	Delphi method	Adults with Long COVID	Clinical	None	85.4	No
Multidisciplinary collaborative consensus guidance statement on the assessment and treatment of neurologic sequelae in patients with PASC	2023	AAPM&R Multi-Disciplinary PASC Collaborative [Melamed et al. (21)]	United States	Mixed public–private insurance	Multidisciplinary expert consensus without GRADE	Adults with Long COVID and neurologic sequelae	Clinical	Institutional funding disclosed, unrelated to this work	81.2	No
Development of a Novel Care Rehabilitation Pathway for Post-COVID Conditions in a Provincial Health System in Alberta, Canada	2022	Alberta Health Services [Pohar Manhas et al. (26)]	Canada	Provincial single-payer system	Health service development / pathway implementation	Adults with Long COVID	Public health and service organisation	None	81.2	No
2022 ACC Expert Consensus Decision Pathway on Cardiovascular Sequelae of COVID-19 in Adults	2022	American College of Cardiology [Gluckman et al. (22)]	United States	Mixed public–private insurance	Expert consensus decision pathway	Adults with possible cardiovascular sequelae	Clinical	None	79.2	No
Swiss Recommendations for the Follow-Up and Treatment of Pulmonary Long COVID	2021	Swiss Society for Pulmonology [Funke-Chambour et al. (23)]	Switzerland	Mandatory social health insurance	Evidence-informed expert consensus	Adults with pulmonary Long COVID	Clinical	None	77.1	No
Clinical Practice Guideline Recommendations for Post-Acute Sequelae of COVID-19	2025	Korea Disease Control and Prevention Agency [Seo et al. (18)]	Republic of Korea	National health insurance (single payer)	GRADE with multidisciplinary expert consensus	Adults with Long COVID	Public health	None	68.8	No
Multidisciplinary collaborative consensus guidance statement on the assessment and treatment of fatigue in PASC patients	2021	AAPM&R Multi-Disciplinary PASC Collaborative [Herrera et al. (36)]	United States	Mixed public–private insurance	Multidisciplinary expert consensus without GRADE	Adults with Long COVID and fatigue	Clinical	None	60.4	No
Evidence-based position paper on physical and rehabilitation medicine professional practice for persons with COVID-19: the European PRM position	2023	UEMS PRM Section [Ceravolo et al. (30)]	Europe (multinational)	Multiple systems	Delphi method	Persons with COVID-19-related functioning limitations	Clinical	None	54.2	No
Evaluation and treatment approaches for neurological post-acute sequelae of COVID-19: consensus statement and scoping review	2023	Global COVID-19 Neuro Research Coalition [Frontera et al. (32)]	Multinational	Multiple systems	Scoping review with multidisciplinary expert consensus	Adults with Long COVID	Clinical	None	52.1	No
Neurological manifestations of post-COVID-19 syndrome: S1-guideline of the German Society of Neurology	2022	German Society of Neurology [Franke et al. (34)]	Germany	Statutory health insurance (multi-payer)	Expert multidisciplinary consensus	Adults with Long COVID and neurological manifestations	Clinical	None	47.9	No
Consensus document of the Mexican Society of Cardiology for post-COVID-19 cardiovascular clinical follow-up	2022	Sociedad Mexicana de Cardiología [Ayala-León et al. (35)]	Mexico	Segmented public–private system	Expert multidisciplinary consensus	Adults with post-COVID cardiovascular sequelae	Clinical	None	41.7	No
Clinical practice guideline for long COVID prevention and treatment	2026	Multidisciplinary Working Group on Long COVID Practice Guidelines [Cao et al. (15)]	Multinational	Multiple systems	GRADE with Delphi consensus	Adults with Long COVID	Clinical	Multiple disclosures reported	40.6	No
The Delphi Method: Developing a Telerehabilitation Practice Guideline for Patients in Indonesia with Long COVID	2024	Universitas Indonesia [Paramita et al. (29)]	Indonesia	National health insurance (single payer)	Delphi method	Adults with Long COVID	Clinical	None	39.6	No
Rehabilitation of Neuropsychiatric Symptoms in Patients With Long COVID: Position Statement	2023	Weill Cornell Medicine [Sacks-Zimmerman et al. (33)]	United States	Mixed public–private insurance	Expert multidisciplinary consensus (position statement)	Adults with Long COVID and neuropsychiatric symptoms	Clinical	None	27.1	No
Multi-disciplinary collaborative consensus guidance statement on the assessment and treatment of mental health symptoms in patients with PASC	2023	AAPM&R Multi-Disciplinary PASC Collaborative [Cheng et al. (31)]	United States	Mixed public–private insurance	Multidisciplinary expert consensus without GRADE	Adults with Long COVID and mental health symptoms	Clinical	Institutional funding disclosed, unrelated to this work	16.7	No

Documents are ordered by AGREE II Domain 3 (Rigor of Development). Rows shaded in green and titles in bold indicate the documents prioritised for the evidence synthesis. Domain 3 scores are standardised percentages computed from the item-level ratings using the formula in the AGREE II user manual; full domain scores for all six domains are reported in Supplementary material.

AAPM&R, American Academy of Physical Medicine and Rehabilitation; ESCMID, European Society of Clinical Microbiology and Infectious Diseases; GRADE, Grading of Recommendations Assessment, Development and Evaluation; PASC, post-acute sequelae of SARS-CoV-2 infection; PRM, Physical and Rehabilitation Medicine; UEMS, Union of European Medical Specialists.

Retaining a superseded version departs from the eligibility rule stated in the registered record and is reported among the protocol deviations rather than as routine application of the original eligibility criteria.

Relevant documents that were assessed at full text but not included are reported in Supplementary material 2.

### Data collection process and data items

Data was extracted by one reviewer using a standardised form and independently verified by a second reviewer. This approach is accepted in systematic reviews of clinical practice guidelines when duplicate extraction is not practical (10). When an item was not explicitly reported in the document, it was recorded as “not reported.” For each document, we extracted: (i) identification details (e.g., title, year, jurisdiction, document type), (ii) stated objectives and scope, (iii) issuing institution or professional society, (iv) clinical questions or domains addressed, and (v) practice recommendations, including (when available) the reported level of evidence, strength of recommendation (e.g., GRADE), or consensus rating. In all cases, we verified whether conflicts of interest were reported.

### Methodological quality and clinical applicability appraisal

All included CPGs and SSs were independently appraised by two reviewers using the AGREE II instrument (19, 20). Following the AGREE II user manual, the six domain scores are reported separately and were not aggregated into a single overall score, since the domains are conceptually independent. Standardisation was performed within each domain separately, following the user manual: for a given domain, the sum of the item ratings obtained across appraisers was rescaled by subtracting the minimum possible sum for that domain and dividing by the difference between the maximum and minimum possible sums for that domain. No summation was performed across domains and no overall guideline score was derived, so the rescaling applies to each of the six domains independently. Documents prioritised for the evidence synthesis were subsequently assessed with the AGREE-REX instrument (20), which comprises nine items grouped into three domains: Clinical Applicability, Values and Preferences, and Implementability. The unit of appraisal was the group of recommendations belonging to each NICE clinical domain within a document, yielding twelve appraisal units across the four prioritised documents. For both instruments, reviewers evaluated all domains and applied the scoring and overall assessment approach recommended in the respective user manuals.

### Effect measures

Not applicable.

### Synthesis methods

We conducted a narrative synthesis of the recommendations and good practice statements reported across the included CPGs and SSs. The synthesis emphasised the degree of concordance across issuing organisations, when recommendations differed across documents, we explored potential reasons for discordance by examining the cited supporting evidence. To minimise double counting and transparently prioritise evidence, we applied the following hierarchical approach: (1) practice recommendations from high methodological-quality CPGs using GRADE; and (2) the most recent, highest-quality SS providing good practice statements. The results are presented using a narrative synthesis approach, structured according to the NICE clinical domains previously outlined in the eligibility criteria section.

Study selection was presented using a PRISMA 2020 flow diagram. Characteristics of included documents were summarised in a descriptive table. Methodological quality and clinical applicability results from AGREE II and AGREE-REX were presented using comparative tables and figures. Extracted recommendations were organised in evidence matrices and grouped according to predefined NICE clinical domains: Care Planning (caring for patients, after assessment in primary care or a multidisciplinary assessment service), Management (care to patients, within the setting of primary care, community environments, or multidisciplinary assessment and rehabilitation services), Monitoring and Follow-up (care to patients across all care settings over time, addressing disease progression and associated monitoring activities, within the setting of primary care, community environments, or multidisciplinary assessment and rehabilitation services), Information Sharing and Continuity of Care (responsible for coordinating and sharing clinical information across services for patients with ongoing symptomatic COVID-19 or post-acute sequelae COVID-19 syndrome (PASC), with the aim of ensuring integrated, continuous, and person-centered care), and Service Organisation (healthcare organisations, service leads, and commissioners responsible for designing, structuring, and delivering care for individuals with ongoing symptomatic COVID-19 or PASC).

The distribution of recommendations by domain was summarised using frequencies and percentages, and the detailed recommendation matrix was provided as Supplementary material.

Diversity in guidance characteristics was examined qualitatively by comparing recommendations within each NICE clinical domain according to document type, issuing organisation, methodological approach, AGREE II/AGREE-REX appraisal, reported certainty of evidence, and clinical scope. Recommendations were considered discordant when they addressed the same clinical issue but differed in the recommended action, target population, intensity of intervention, setting, or implementation conditions. Discordances were summarised narratively and, when applicable, reported in evidence matrices. Also, disagreements were solved by consensus between two reviewers, with arbitration by a third reviewer when needed (see Supplementary material 2 in the sheet “Complete”).

Sensitivity analyses were not conducted because the review did not involve quantitative synthesis or meta-analysis. The robustness of findings was addressed through methodological appraisal (AGREE II and AGREE-REX) and prioritisation of higher-quality guidance documents.

### Reporting bias assessment

This review does not estimate effect parameters, so a quantitative assessment of publication bias, such as a funnel plot or a statistical test for small-study effects, was neither applicable nor performed. Reporting bias may nevertheless affect the review indirectly, in two ways. First, the systematic reviews and primary studies underpinning the recommendations of the included documents may themselves be subject to publication and outcome reporting bias; where that is the case, the bias is carried into the recommendation without being visible at the level of the guidance document, and few of the included documents reported having assessed this risk in their own evidence base. Second, the retrievability of guidance is uneven: documents issued by health services rather than published in indexed journals are less likely to be retrieved, so the type and frequency of the recommendations reported here may under-represent guidance on service organisation. The distribution of recommendations across domains should therefore be read as a description of the retrieved corpus rather than of the guidance produced worldwide.

Consistent with the above, we relied on the quality and risk-of-bias assessments reported by the developers of the included guidance documents, rather than conducting an independent evaluation ourselves. This approach was adopted to maintain consistency with the original publications and to avoid introducing additional subjective judgments beyond those already documented in the source articles.

### Certainty assessment

We extracted the reported certainty level of evidence and the strength of each recommendation; when not provided, we coded the item as “not reported” or “not applicable,” as appropriate. For this systematic review, the certainty of the evidence and the strength of the recommendations were strictly extracted and reported exactly as presented in each original CPGs. No additional or independent systematic framework (such as a separate GRADE assessment) was applied by the authors to re-evaluate these ratings.

To ensure the Rigor and reproducibility of the review, the eligibility assessment, document selection, and data extraction processes were performed independently by two primary reviewers. Methodological quality and clinical credibility were systematically evaluated using standardised instruments. Recommendations were assigned to one of five NICE-based domains according to their primary clinical purpose. When a recommendation addressed more than one domain, it was classified according to the main action required from the care team. Disagreements in classification were resolved by consensus between two reviewers, with arbitration by a third reviewer when needed (see Supplementary material 2 in the sheet “Complete”). The entire process, including tracking, consensus documentation, and final data synthesis, was fully managed and systematised using Microsoft Excel.

## Results

### Selection of guidance documents

The systematic literature search was conducted across 2 databases and 5 registers, initially identifying 5,910 records. A further 85 records were identified through alternative methods (Trip *n* = 79, Guidelines International Network *n* = 1, NICE *n* = 2, SIGN *n* = 2, and the American College of Physicians *n* = 1; the WHO portal and the Spanish, Mexican and United States government health portals returned no eligible records). After removing 1,581 duplicates, 4,329 records from databases and registers were screened by title and abstract, of which 4,229 were excluded, leaving 100 reports sought for retrieval; 14 could not be retrieved, so 86 reports were assessed for eligibility. In the other-methods strand, all 85 records were sought for retrieval, 6 could not be retrieved, and the 79 reports assessed for eligibility were all excluded as duplicates of documents already retrieved, so this strand contributed no documents to the review. Of the 86 reports assessed for eligibility in the databases and registers strand, 36 were retained after independent full-text assessment by both reviewers; the remainder were excluded because they were not a clinical practice guideline or scientific statement, were not focused on adults with Long COVID, did not include an intervention, or did not contain recommendations. Of the 36 documents retained, 17 were not carried forward because they were published in languages other than English or Spanish or were superseded versions of guidance already represented in the corpus, leaving 19 documents for AGREE II appraisal. One superseded version, Kim et al. (17), was retained by exception and appraised alongside its 2025 update (see Methods, Documented exceptions, and Registration and protocol, Deviation 9).

The updated search performed on 2 September 2026 identified 298 additional records after removal of records already screened. Of these, 296 were excluded at title and abstract screening and 2 were assessed at full text, from which one document (15) met the eligibility criteria and underwent AGREE II appraisal. The appraised corpus therefore comprised 20 guidance documents (see Figure 1).

**Figure 1:**
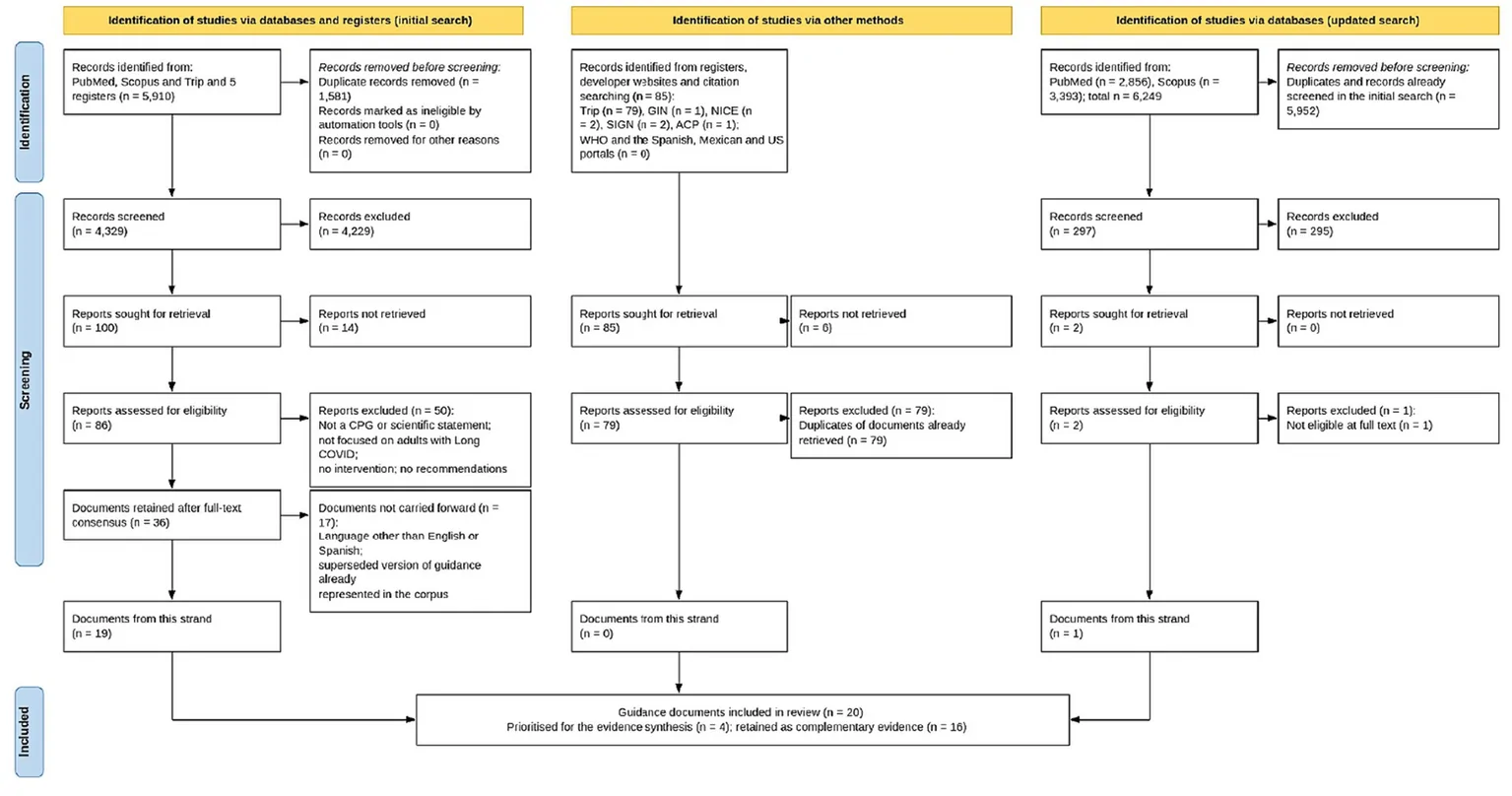
PRISMA flow diagram.

### Application of the prioritisation criteria

Of the 20 appraised documents, eleven scored below 70% in AGREE II Domain 3, Rigor of Development; ten of these were not carried forward at Step 1 and one, Fine et al. (16), was retained under the documented exception reported in the Methods. Of the nine documents meeting the quality threshold, three were not carried forward at Step 2 because their thematic scope was restricted to a single clinical manifestation: neurological sequelae (21), cardiovascular sequelae (22) and pulmonary Long COVID (23). Of the six documents reaching Step 3, three were not carried forward because they contributed no clinical domain or recommendation type not already represented in the prioritised set: Yelin et al. (24), Nurek et al. (25) and Pohar Manhas et al. (26). Four documents were therefore prioritised for the evidence synthesis, one of them under an explicit exception, and they contributed 99 of the 510 extracted recommendations.

The four prioritised documents contribute complementary areas and recommendation types. NICE (27) (Rigor of Development 85.4%) was the only document in the corpus reporting an evidence-based, GRADE-compatible development process; it covers the full care pathway and is the sole source of the seven recommendations on Sharing Information and Continuity of Care and of four of the six Service Organisation recommendations in the corpus, both of them domains not addressed by any other prioritised document. Kim et al. (17) (95.8%), an expert multidisciplinary consensus developed for a single-payer national health insurance system, contributes clinical recommendations concentrated in Care Planning and Management. Giuliano et al. (28) (79.2%), a multidisciplinary expert consensus without GRADE issued for a tax-funded national health service, combines clinical recommendations with service-organisation and rehabilitation guidance. Fine et al. (16) (62.5%), a multidisciplinary expert consensus without GRADE, is the sole source of recommendations on the assessment and management of cognitive symptoms, a domain otherwise addressed only through general screening statements.

Yelin et al. (24), the ESCMID rapid guideline for the assessment and management of Long COVID, achieved the highest Rigor of Development score in the corpus (97.9%) and satisfied Steps 1 and 2, but was not carried forward at Step 3. Its recommendations address the initial assessment and general management of the condition, that is, clinical recommendations within Care Planning and Management, and that scope was already covered by NICE (27) and Kim et al. (17), the former reporting a GRADE-compatible development process. Whereas each prioritised document contributed a clinical domain or a recommendation type not otherwise represented in the set, Yelin et al. (24) would have contributed overlapping recommendations without broadening the thematic coverage of the synthesis. Its recommendations were therefore retained as complementary evidence, are reported in the recommendation matrix, and contribute to the comparison of discordant recommendations in Care Planning.

### Characteristics of the guidance documents

The 20 included guidance documents comprise clinical practice guidelines, consensus statements, position papers, and Delphi-based recommendations published between 2021 and 2026, all addressing the assessment and management of PASC in adults across clinical and public health settings (15–18, 21–36). The documents were developed by national and international organisations as well as multidisciplinary research teams, and cover a broad range of sequelae including neurological, cardiovascular, cognitive, fatigue, pulmonary, and mental health symptoms.

Most were produced using expert multidisciplinary consensus methods, with some employing the GRADE methodology (18), Delphi processes (25, 29, 30) or rapid guideline frameworks (24, 27). Notable contributors include the American Academy of Physical Medicine and Rehabilitation (AAPM&R) Multi-Disciplinary PASC Collaborative, which produced multiple consensus statements on fatigue (36), cognitive symptoms (16), and neurological sequelae (32), as well as Frontera et al. (32), Cheng et al. (31), Giuliano et al. (28), Kim et al. (17), Manhas et al. (26), Sacks-Zimmerman et al. (33), the German Society of Neurology (34), the Mexican Society of Cardiology (35), the American College of Cardiology (22), and the Swiss Society for Pulmonology (23). Most of the guidance documents reported no conflicts of interest, apart from select AAPM&R collaborative statements in which some authors disclosed institutional funding unrelated to the specific manuscripts (see Table 1).

For clinical practice guidelines that have undergone sequential updates or revisions by the same issuing organisation (e.g., National Institute for Health and Care Excellence [NICE]), multiple publications addressing the same core pathway were treated as a single unified guideline unit. Specifically, NICE NG188 (2020) and its updated iteration, NICE (27), were evaluated and synthesised as a single, consolidated document representing the updated NICE clinical recommendations. AGREE II evaluations reflect the composite evidence base of the consolidated version.

Sequential versions were handled in two different ways in this review, and the distinction is made explicit here. Where successive publications addressed the same core pathway and the later one incorporated the earlier, as with NICE NG188 (2020) and NICE (27), they were appraised and synthesised as a single consolidated unit. Where successive versions differed substantially in reporting quality, as with Kim et al. (17) and Seo et al. (18), each version was appraised separately and both are reported in Table 1, and only the earlier version was carried forward to the prioritised set, for the reasons given in the Methods.

### Methodological quality and applicability appraisal

The unit of appraisal for AGREE II was the guidance document. Where an issuing body had published successive documents addressing the same core pathway and the later publication incorporated the earlier one, the versions were appraised as a single consolidated unit; this applied only to NICE NG188 (2020) and its 2024 update. Sequential versions that differed substantially in reporting quality were appraised separately, as described for Kim et al. (17) and Seo et al. (18). The appraised corpus therefore comprises 20 appraisal units, and this number is used consistently in the text, in Table 1 and Figure 2.

**Figure 2:**
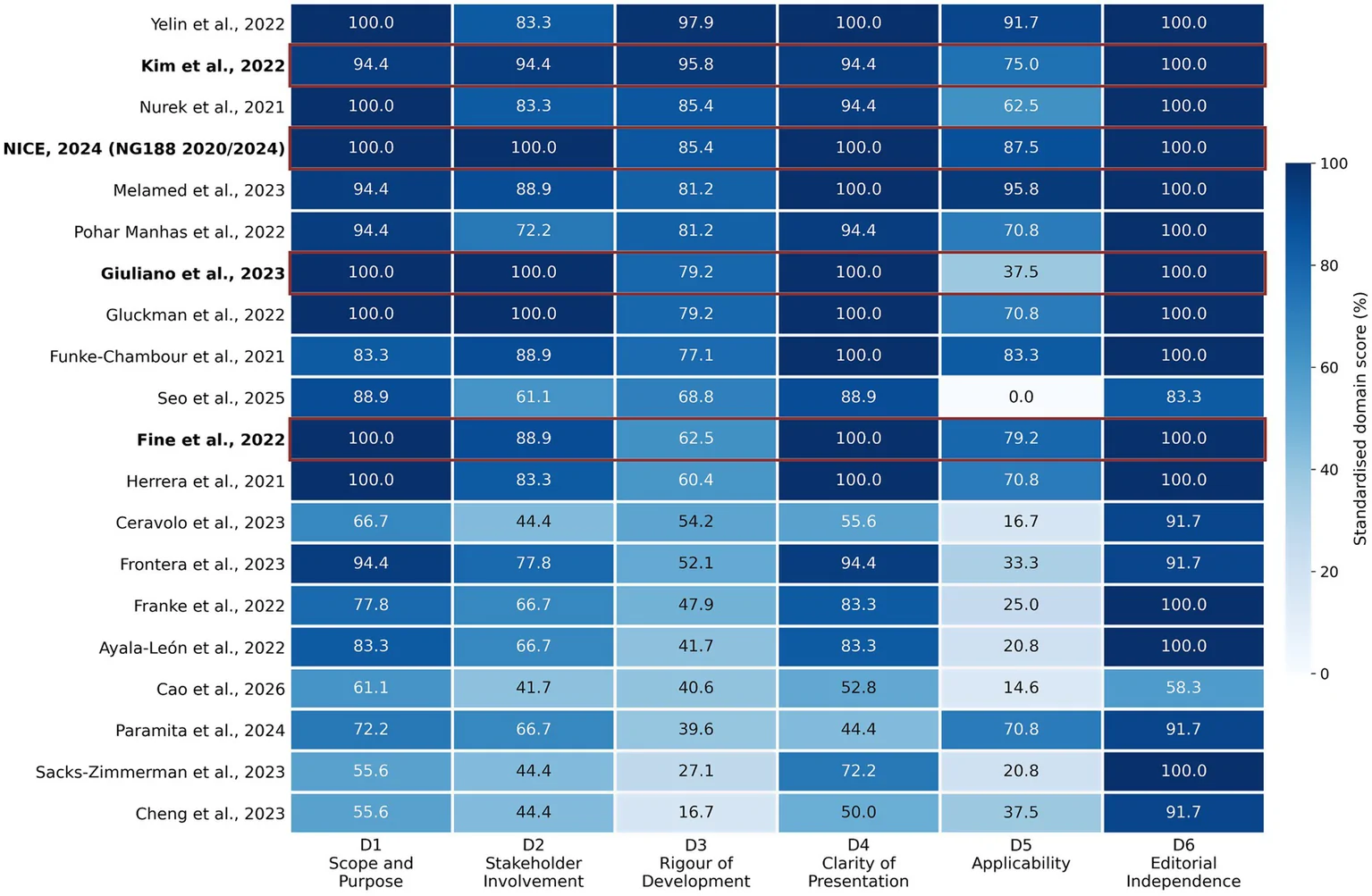
AGREE II standardised domain scores across the appraised guidance documents (*n* = 20). Methodological quality and applicability of the appraised documents.

The included guidance documents were appraised using the AGREE II and AGREE-REX instruments. These instruments assess the methodological quality and reporting of guideline development and the quality and applicability of recommendations, respectively, and are not conventional risk-of-bias tools. AGREE II was selected because it provides a validated, internationally recognised framework for evaluating the methodological Rigor and transparency of guideline development across six domains: scope and purpose, stakeholder involvement, rigor of development, clarity of presentation, applicability, and editorial independence. While AGREE-REX was used to complement this assessment by specifically evaluating the clinical credibility and applicability of the recommendations, (addressing dimensions not fully captured by AGREE II, such as the values and preferences of target users, implementability, and applicability to clinical decision-making contexts). The combined use of both instruments allowed for a more comprehensive evaluation of both the developmental quality and the practical relevance of each guideline, thereby strengthening the reliability of the inclusion criteria applied in this review.

### Methodological quality

Domain scores varied substantially across the 20 documents. Rigor of Development ranged from 16.7 to 97.9% and Applicability from 0.0 to 95.8%, whereas Editorial Independence was high throughout (median 100%). Comparing the four prioritised documents with the remaining sixteen, the highest scores among the prioritised documents were observed in Editorial Independence (100%), Scope and Purpose (98.3%), and Clarity of Presentation (98.6%), reflecting strong methodological transparency and clear formulation of recommendations. Stakeholder Involvement scored 96%, while Rigor of Development reached 80.7%. The lowest-scoring domain among the prioritised documents was Applicability (69.8%), although it still exceeded the corresponding mean among the documents retained as complementary evidence (50%). Rigor of Development among the prioritised documents ranged from 62.5 to 95.8%. In all six domains, the prioritised documents scored higher on average than those retained as complementary evidence, with the largest differences in Stakeholder Involvement (+21 points) and Rigor of Development (+19 points; see Figure 2).

### Clinical applicability

AGREE-REX was applied to the four documents prioritised for the evidence synthesis, after prioritisation, and therefore did not contribute to their selection. The unit of appraisal was the group of recommendations belonging to each NICE clinical domain within a document, yielding twelve appraisal units. Results are reported for the three domains of the instrument. Standardised domain scores were 86.1% for Clinical Applicability (range 72.2–100), 87.8% for Values and Preferences (range 75.0–95.8), and 91.7% for Implementability (range 83.3–100). At document level, NICE (27) scored highest across all three domains (91.1, 95.0 and 93.3%), followed by Giuliano et al. (28) (85.2, 86.1, 91.7%) and Fine et al. (16) (88.9, 81.2, 91.7%); Kim et al. (17) scored lowest in Clinical Applicability (72.2%) and Values and Preferences (79.2%).

At item level, Purpose and Values and Preferences of Target Users were the strongest items (mean 6.7 of 7), followed by Applicability to Target Users (6.6). The weakest item across all appraisal units was Applicability to Patients and Populations (5.8), with Values and Preferences of Patients and Populations and of Policy and Decision-Makers next lowest (6.0 each). This pattern indicates that the prioritised documents are more clearly directed at clinicians than at patients or decision-makers (see Figure 3).

**Figure 3:**
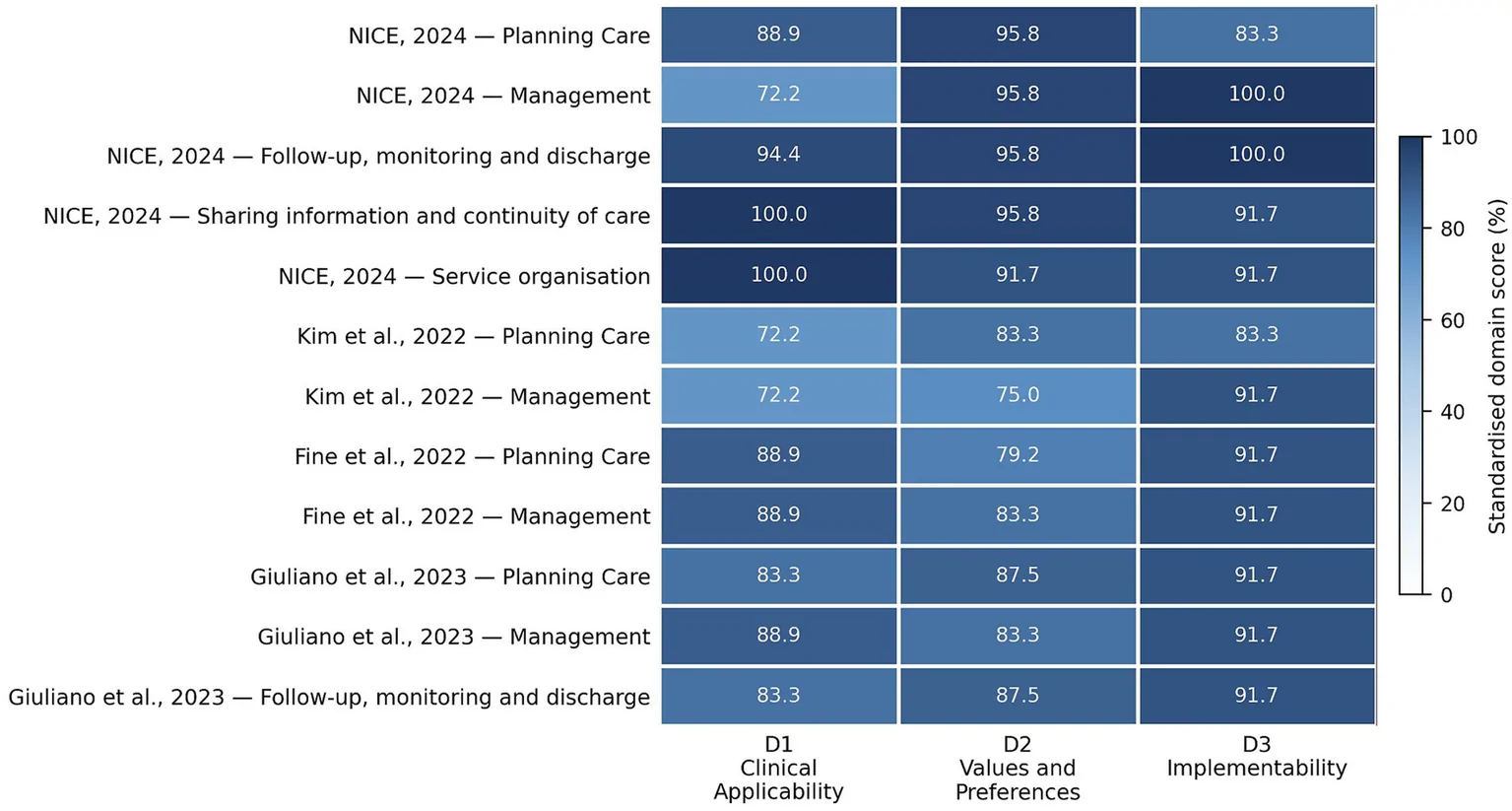
AGREE-REX standardised domain scores by appraisal unit (*n* = 12). The unit of appraisal is the group of recommendations belonging to each NICE clinical domain within a document, across the four prioritised documents.

Documents are ordered by Rigor of Development; those prioritised for the evidence synthesis are outlined and shown in bold. The six domains are conceptually independent and are not aggregated into a single score.

Both figures replace the radar plots submitted previously, which superimposed multiple documents on a single axis and included an aggregate score alongside the six domains.

Source data for Figures 2,3, including the item-level appraisal ratings, are provided in Supplementary material.

The clinical domains applied to each guidance document were derived from the NICE guideline (27).

From the 19 documents appraised in the initial search, a total of 510 recommendations were extracted, distributed across the five NICE-derived clinical domains. The domain with the highest number of recommendations was Management with 323 (63.3%), followed by Care Planning with 151 (29.6%), Follow-up, Monitoring and Discharge with 23 (4.5%), Sharing Information and Continuity of Care with 7 (1.4%), and Service Organisation with 6 recommendations (1.2%). These counts are presented descriptively and should not be read as a measure of how developed each clinical domain is, because the number of recommendations depends strongly on how each document structures and subdivides its statements. A single document, Frontera et al. (32), contributed 142 of the 510 recommendations, that is 27.8% of the total (see Table 2). The document added by the updated search (15) contains 10 further recommendations, which are recorded in the supplementary recommendation matrix as complementary evidence and are not included in the 510 recommendations reported in Table 2.

**Table 2:** Recommendations based on dimensions.

Domain	Number of recommendations	Percentage
Care planning	151	29.6%
Management	323	63.3%
Follow-up, monitoring and discharge	23	4.5%
Sharing information and continuity of care	7	1.4%
Service organisation	6	1.2%
Global	510	100%

The 99 recommendations contributed by the prioritised documents are distributed as follows: Care Planning 43 (43.4% of the 99), Management 34 (34.3%), Follow-up, Monitoring and Discharge 11 (11.1%), Sharing Information and Continuity of Care 7 (7.1%), and Service Organisation 4 (4.0%; for more information see “Supplementary material 2” in the sheet “Number of recommendations”).

Summary of recommendations are presented in Supplementary material 3. Discordant recommendations. Comparison of recommendations addressing the same clinical question across documents identified four discordances, one in Management and three in Care Planning. In Management, documents differ on the prescription of exercise in patients with post-exertional malaise: Seo et al. (18) recommend pacing and symptom-titrated activity while explicitly avoiding graded exercise programmes, and Fine et al. (17) recommend an initial phase of convalescence followed by careful pacing, whereas Ceravolo et al. (30) recommend tailored physical exercise training without conditioning it on prior screening for post-exertional malaise, and Melamed et al. (21) recommend regular activity as tolerated in patients who have already returned to daily activities. In Care Planning, documents differ on the extent of laboratory testing at initial assessment, with NICE (27) conditioning blood tests on clinical indication and Fine et al. (17) specifying a broader defined panel; on the timing and threshold for chest radiography, offered by NICE only at 12 weeks and when clinically indicated, at an early stage by Fine et al., and in the presence of cough by Seo et al.; and on what is offered between weeks four and twelve, where NICE initiates self-management support and Yelin et al. (24) assess case by case. No discordances were identified in Follow-up, Information Sharing or Service Organisation, domains in which most recommendations derive from a single document, so that the absence of discordance does not indicate independent corroboration. The basis for assessing concordance therefore varies markedly across domains: several documents contribute to Care Planning and Management, whereas NICE (27) is the sole source of the seven recommendations in Information Sharing and Continuity of Care and contributes four of the six in Service Organisation. Statements about agreement across issuing organisations apply to the first two domains and not to the remainder. The full discordance matrix, including the position of each document with its original grading and the apparent reason for each difference, is provided as Supplementary material.

### Evidence synthesis

For each domain, recommendations were interpreted considering the number of source guidance documents issuing a concordant recommendation (that is, the number of CPGs or SSs, not the number of primary studies cited within them), the methodological quality of those source documents, the certainty or strength of recommendation reported by each source, and the applicability of the recommendation according to AGREE-REX where available. See more in Supplementary material 2.

### Planning care

International guidelines differentiate between two distinct clinical categories of PASC symptoms: ongoing symptomatic COVID-19, defined as symptoms persisting between 4 and 12 weeks without an alternative diagnosis (27), and PASC, defined as symptoms extending beyond 12 weeks, typically associated with a chronic trajectory requiring a multidisciplinary rehabilitation approach (17). Characterised also by manifesting as fluctuating symptom clusters that require specific evaluation criteria (27, 28). An optimal clinical approach demands a multidimensional and comprehensive assessment (16, 28), initiating self-management support at week four and referring to specialised services as necessary (27). Finally, management must focus on holistic care plans and shared decision-making, structured under coordinated care pathways between primary care and multidisciplinary clinics (27, 28).

### Management

Initial consultations should prioritise shared decision-making and the use of validated screening tools to ensure a holistic, patient-centered assessment covering physical, cognitive, and psychiatric symptoms, as well as functional status (27). It is essential to personalise rehabilitation through documented plans that incorporate patient-led goals, specific interventions, and monitoring strategies for fluctuating symptoms, while adhering to recognised rehabilitation standards to optimize outcomes (16, 17, 27). Effective care planning and continuity of follow-up are defined by six key elements: individualised pathways, support for returning to work or studies, self-management, multidisciplinary rehabilitation, care for vulnerable populations, and long-term continuity (28).

### Follow-up, monitoring and discharge

Baseline data collected during the initial assessment through standardised performance indicators is essential for monitoring functional changes during follow-up appointments (27, 28). PASC monitoring should be prioritised by primary care or community services for high-risk groups, while hospitalised patients require specialist follow-up to evaluate complications and persistent symptoms. Consistent with shared decision-making, the frequency and nature of this monitoring must be personalised, balancing the patient’s specific needs and symptoms with service availability and accessibility (27).

### Sharing information and continuity of care

Effective management requires continuous information exchange with patients, families, and caregivers regarding symptom self-management, recovery expectations, and vaccination (27). To ensure continuity of care, patients should be assigned a care coordinator and granted access to their personalised care plans, which facilitate the comparison of longitudinal data and must be shared across the multidisciplinary team. Furthermore, this information should be provided in age-appropriate and accessible formats to empower patients to actively participate in shared decision-making regarding their treatment (27).

### Service organisation

Coordinated care and local pathways should be established among multidisciplinary services such as rehabilitation, assessment and mental health, provided by specialists, both at primary and community care. Considering the range of symptoms the patient has; these multidisciplinary services should be guided by a qualified professional supported by a proper specialist and skilled in managing daily fatigue and respiratory symptoms and considering further mastery that might be needed depending on the age and individual symptoms. Also, local needs and resources should be considered (27).

## Discussion

### Methodological quality of the appraised guidance

The primary aim of this review was to appraise the methodological quality and clinical applicability of the guidance available for adults with Long COVID, and the appraisal is therefore where the discussion begins. Quality was highly uneven across the 20 appraised documents: Rigor of Development ranged from 16.7 to 97.9% and Applicability from 0.0 to 95.8%, while Editorial Independence was high throughout (median 100%). The dispersion follows a consistent profile rather than a random one, with high scores in Scope and Purpose, Clarity of Presentation and Editorial Independence alongside markedly lower scores in Rigor of Development and Applicability. Most of the available guidance is therefore clearly written and transparently sponsored, but the process linking evidence to recommendations, and the guidance offered for putting those recommendations into practice, are reported far less consistently. Only one document, NICE (27), reported a GRADE-compatible evidence-to-decision framework, so for most of the corpus the certainty of the underlying evidence cannot be traced from a recommendation back to the evidence that supports it. The four prioritised documents scored higher than the remaining sixteen in all six domains, with the largest differences in Stakeholder Involvement and Rigor of Development, which confirms that the prioritisation step discriminated on the dimension it was designed to discriminate on.

### Clinical applicability of the prioritised recommendations

Applicability was the weakest AGREE II domain among the prioritised documents (68%), even though it exceeded the mean of the documents retained as complementary evidence (50%). The AGREE-REX appraisal locates that weakness more precisely. Standardised domain scores were high overall, at 86.1% for Clinical Applicability, 87.8% for Values and Preferences and 91.7% for Implementability, but the item-level pattern was consistent across the twelve appraisal units: Applicability to Patients and Populations was the weakest item (5.8 of 7), followed by the Values and Preferences of patients and populations and of policy and decision-makers (6.0 each), whereas the items concerning target users, that is clinicians, scored highest. The prioritised guidance is thus written for the clinician rather than for the patient who must follow it or the decision-maker who must fund and organise the services it presupposes. This has a direct consequence for adaptation: recommendations can be transferred to another health system with reasonable confidence as to what should be done clinically, but with little explicit information on patient values, resource implications or implementation barriers, which are precisely the inputs an evidence-to-decision framework requires.

### Comparison with previous appraisals

Three previous reviews have appraised Long COVID guidance and provide a reference point for our findings. Marshall-Andon et al. (7) reviewed post-COVID-19 syndrome rehabilitation guidelines using AGREE II, AGREE-REX and AGREE-HS with independent duplicate assessment, but restricted the scope to rehabilitation. Luo et al. (8) appraised five international guidelines with AGREE II and rated one as high quality, three as moderate and one as low. Luo et al. (9) extended that work to three recent national documents from Australia, the United States and Canada, all of which received overall global ratings of 5 to 6 out of 7.

Our findings are consistent with this literature in showing wide variation in methodological quality, and they extend it in two respects. First, the appraised corpus is broader, covering the full care pathway rather than a single clinical area, and the synthesis operates at the level of the individual recommendation rather than the document. RigorSecond, the domain profile we observed, with high scores for Scope and Purpose, Clarity of Presentation and Editorial Independence alongside markedly lower scores for Rigor of Development and Applicability, mirrors the pattern reported in appraisals of guidance in unrelated clinical fields, suggesting that it reflects a general feature of guideline development rather than a characteristic of Long COVID guidance specifically.

One methodological difference deserves comment. Luo et al. (9) report inter-rater reliability for their appraisals (ICC 0.89 to 0.99), whereas only consensus scores were retained in the present review, so no equivalent statistic could be computed. Prospective documentation of appraiser agreement should be standard practice in reviews of this kind.

### Clinical content of the recommendations

The content of the recommendations converges considerably more than their methodological quality does, and the clinical picture it describes is consistent with the wider literature on the burden of Long COVID summarised below.

Coronavirus Disease 2019 (COVID-19) is caused by the severe acute respiratory syndrome coronavirus 2 (SARS-CoV-2). Over the past 4 years, there have been more than 774 million confirmed cases worldwide (37). While some patients develop COVID-19 pneumonia, approximately 90% of people infected with SARS-CoV-2 experience only mild initial respiratory symptoms and do not require hospitalisation. However, many people experience symptoms that persist for more than 12 weeks after the onset of COVID-19, a condition now referred to as long COVID (38).

In the United States, it has been estimated that people living with long COVID are more likely to report a history of recent unemployment, financial difficulties, anxiety, and depression (39). These findings have been corroborated, highlighting the multidimensional nature of the impact of long COVID and the social vulnerability it can entail for people living with this condition, particularly those belonging to minority groups (40–42). Other studies have also identified increased demand for medical care following diagnosis (43) and disparities in access (44). Similarly, healthcare providers report lacking the specific tools needed to care for and treat this population, with significant gaps in knowledge among different providers (45).

In our systematic review, clinical practice guidelines emphasise the importance of shared decision-making with the patient, which should take place following the initial evaluation, as well as the development of a collaborative care plan with the patient. This shared decision-making process aims to highlight the patient’s individual circumstances, including their goals, preferences, and social support, as well as the frequency of follow-up visits, the professionals involved in the process, and the type of service provided. As a result, decisions will be guided by the patient’s needs and symptoms, as well as their sociocultural context. In addition, this would provide disadvantaged communities with easier access to health services, both in terms of medical care and disease awareness. Both assessment and management must consider a comprehensive evaluation and patient-centered care (27).

The impact on work performance has also been highlighted in various external evidence studies. Patients living with long COVID report difficulties adapting to their workplaces and even feelings of guilt over poor performance, while their supervisors report challenges related to sick leave policies and a lack of information on how to interact with colleagues living with this condition in the workplace (46). Furthermore, studies have reported that, in the workplace, patients with long COVID are often diagnosed with burnout and are prone to stigmatisation and the “psychologisation” of their symptoms (47). Similar findings have been reported regarding the impact of long COVID on academic performance (48).

In fact, both in external evidence studies and in the synthesis carried out it is established that, when sharing information with the patient and ensuring continuity of care, one must specify the communication channel the patient can use if they encounter difficulties in resuming and readjusting to their daily activities and routine (27). Furthermore, returning to work or school is considered a key factor to consider when developing a care plan and ensuring continuity of follow-up, as well as when assessing potential long-term effects that the patient may experience in various aspects of daily life (16, 28).

Sharon and Matthies-Boon (49) have highlighted the discrepancy between the solidarity shown toward COVID-19 patients during the pandemic and the lesser solidarity shown toward patients living with the aftereffects of long COVID. The authors highlight two key factors to explain this decline in solidarity: (1) the lack of institutional solidarity versus individual responsibility regarding the infection, and (2) the invisibility of long COVID due to “institutional burnout,” which led to a cessation of monitoring the infection and its consequences.

Giuliano et al. (28) highlight the importance of defining and establishing the roles of healthcare providers in these cases, including primary care, where patients first seek treatment, and where patients with less complex symptoms are treated, while those with more complex clinical conditions are referred to specialised centers. In addition, they play a key role in the multidisciplinary team that evaluates and treats patients with long COVID, not only in medical settings but also in rehabilitation, monitoring, and at the community level, often serving as the link between the patient and the specialist responsible for their specific case (27). Both the definition and the function of these roles are mentioned as key elements in our systematic review.

The multifaceted nature of the effects of long COVID, the debilitating nature of the condition due to fatigue, neurological, and emotional symptoms, and the lack of tools and “institutional support” pose a challenge to providing adequate and comprehensive care for people living with this condition (50). Consequently, it is necessary to move beyond the narrow focus on individual patient care and rehabilitation and instead establish a comprehensive care framework that addresses various aspects of functionality and quality of life, involving the key stakeholders in the patient’s daily life: patients, caregivers, and representatives from postsecondary educational institutions and occupational safety and health organisations.

Clinical practice guidelines not only provide guidance on how to make an accurate diagnosis and conduct a thorough assessment, but they also outline key considerations that must be considered when providing comprehensive care for patients with long COVID. These points include and emphasise aspects such as active patient involvement at every stage of the process, in order to take into account, the patient’s specific circumstances, such as limitations and goals; they also highlight the importance of a multidisciplinary team that maintains constant communication for the patient’s evaluation, treatment, and follow-up, with an emphasis on primary care, which serves as a bridge between the patient, specialists, and the community.

### Strengths

This review followed a transparent methodology, including prospective registration in PROSPERO before formal searching began, adherence to the PRISMA 2020 statement, and appraisal of all eligible documents with AGREE II, complemented by AGREE-REX for the prioritised subset. The search spanned bibliographic databases, international guideline repositories, national health portals and the eCOVID-19 Living Map of Recommendations, and was updated during peer review. Prioritisation followed criteria derived from the registered protocol and applied to the AGREE II domain scores, which are reported in full for every appraised document so that the effect of the prioritisation can be inspected, and item-level appraisal ratings are provided as Supplementary material. Beyond methodological rigor, this review holds value as an input for GRADE-ADOLOPMENT, the process by which recommendations from existing guidelines are adopted, adapted or developed *de novo* for a target health system. It identifies and appraises the available guidance, which is the first step of that process, but it does not evaluate implementation in any specific setting, and the recommendations it consolidates require contextual adaptation before use. The synthesis covers a broad and clinically meaningful range of domains from care planning and multidisciplinary rehabilitation to return-to-work support and continuity of care, reflecting the multidimensional nature of Long COVID. The inclusion of up-to-date evidence, with references as recent as 2026, further positions this document as a timely resource for clinicians and policymakers navigating a rapidly evolving evidence landscape. A further strength is the scope of the appraisal itself: every eligible document was appraised with AGREE II and the prioritised subset with AGREE-REX, with both appraisals reported at domain and item level. A methodological appraisal of this breadth is uncommon in the literature on Long COVID guidance, where previous reviews have been restricted to a single clinical area or to a small number of documents.

### Limitations

Several limitations of this review warrant consideration. The restriction of eligibility to documents published in English or Spanish, specified in the registered protocol, may have excluded relevant guidance from regions where other languages predominate. Geographical representativeness is limited in a second and more consequential way: of 332 adult post-COVID recommendations indexed in the eCOVID-19 RecMap, only 11 originate from Latin America and the Caribbean, and none from Colombia. The evidence base underpinning this synthesis is therefore drawn almost entirely from high-income settings. Consequently, our conclusions should be interpreted as highly representative of specific (18) directly. Therefore, while some recommendations are universally agreed upon, the specific clinical protocols to achieve them still reflect varying degrees of empirical evidence.

The application of the AGREE-REX instrument also presents specific constraints, as appraisals were conducted without full knowledge of the normative and health system context in which each guideline was originally developed, which may have affected the accuracy of applicability ratings. This information gap means that our clinical applicability ratings must be interpreted as standardised theoretical potential rather than guaranteed real-world feasibility. Moreover, although AGREE-REX was applied to the group of recommendations belonging to each NICE clinical domain rather than to the document as a whole, the appraisal unit remains a group of recommendations and not an individual recommendation, so variation between individual recommendations within the same domain is not captured. The granularity of the appraisal is also uneven across domains: the units corresponding to Sharing Information and Continuity of Care and to Service Organisation derive from a single document, so their ratings describe that document rather than the state of the guidance in those domains.

Finally, although an extensive search was conducted, it cannot be guaranteed that all relevant CPGs and SSs were identified. The retrievability of guidance is itself uneven: national guideline repositories are not permanent, and the Canadian Medical Association CPG Infobase, formerly a principal route of access to Canadian guidance, was withdrawn in December 2023. The evidence base for Long COVID management also continues to evolve rapidly. The searches were updated to 2 September 2026 during peer review, which added one further guidance document to the appraised corpus, but some recommendations may already be subject to revision as new clinical trials emerge. Furthermore, many of the included CPGs and SSs did not employ the GRADE methodology, which limits the ability to systematically assess the certainty of the underlying evidence. Several further limitations arise from the conduct of this review itself. The evidence synthesis was restricted to a prioritised subset of the eligible corpus, and although the prioritisation criteria were prespecified, their application involved judgement, particularly in assessing thematic scope. Seventeen documents retained at full-text screening were not appraised because of the language restriction, which may have excluded relevant national guidance, and guidance issued by health services rather than published in journals is less likely to be indexed and may therefore be underrepresented, which is a plausible contributor to the small number of recommendations retrieved in the Service Organisation domain. The included documents were heterogeneous in type, ranging from evidence-based guidelines to expert consensus statements, and no independent assessment of the certainty of the underlying evidence was conducted. Inter-rater reliability was documented for full-text screening but not for the AGREE II and AGREE-REX appraisals, for which only consensus scores were retained. Finally, guidance on Long COVID continues to change rapidly, and high AGREE scores indicate well-reported guideline development rather than high-certainty clinical evidence. The geographical concentration of available guidance is itself a limitation of the evidence base rather than of this review: at the time of writing the eCOVID-19 Living Map of Recommendations recorded 332 post-COVID-19 recommendations for adults worldwide, of which 11 originated in Latin America and the Caribbean and none in Colombia.

### Implications for clinical practice

To apply the care of Long COVID, the transition to a structured and multidisciplinary model led by primary care and with local tools to manage mild to moderate cases and coordinate the timely referral of complex cases is essential. Additionally, to mitigate burnout in the workplace and academic settings, care plans should integrate communication channels that are specific and standardised and that allow patients to report difficulties in real time during their gradual return to daily routines.

### Implications for health policy

To strengthen health policies despite Long COVID, institutional burnout must be counted by providing funding for specialised clinics and long-term surveillance, supported by sick leave policies and institutional guidelines, or existing high-quality and conflict-free recommendations, that prevent the stigmatisation of symptoms in work and academic settings.

### Implications for future research

Future research on Long COVID should prioritise comparative effectiveness studies to assess which service delivery models, between centralised clinics and decentralised primary care networks, optimize clinical outcomes and resource utilization. Along with this, the barriers that delay smooth transitions between acute care, rehabilitation, and occupational health should also be investigated. And another subject to prioritised in future research is the development of prospective longitudinal cohorts that establish protocols that are evidence-based and long-term follow-up, this by defining specific metrics and frequencies on functional recovery and quality of life.

## Conclusion

Guidance for adults with Long COVID is abundant but methodologically uneven, and the unevenness is concentrated in the two dimensions that matter most for use: the rigor with which evidence is linked to recommendations, and the applicability of the resulting guidance. Only one of the 20 appraised documents reported a GRADE-compatible evidence-to-decision framework, and even the four documents of highest methodological quality address clinicians far more clearly than patients or decision-makers. The existing corpus should therefore be treated as a source of well-formulated clinical content that requires formal contextual adaptation, rather than as guidance that can be implemented directly in a new health system. For guideline developers, the priority is the explicit reporting of evidence-to-decision processes, of the values and preferences of patients, and of the resources and barriers that implementation entails. For reviewers and adapters, appraisal at the level of the individual recommendation, rather than of the document as a whole, is what makes the available guidance usable.

## Data Availability

The original contributions presented in the study are included in the article/Supplementary material, further inquiries can be directed to the corresponding author.

## Footnotes

1 covid19.recmap.org.

2 can-pcc.recmap.org.

## Author’s note

This systematic review was registered in the PROSPERO database (Registration No. CRD420261324906). A full protocol was not written; the registered PROSPERO record constitutes the protocol and is provided as Supplementary material. The review departed from the registered record in the following respects. Deviation 1, sources: the protocol specified MEDLINE and Scopus with forward citation searching, whereas the review additionally searched Trip, five international guideline repositories, national health portals and the eCOVID-19 Living Map of Recommendations. Deviation 2, search dates and syntax: the protocol set a cut-off of 24 February 2026; following peer review the PubMed and Scopus searches were corrected and re-executed on 2 September 2026, and the additional records retrieved were screened under the same eligibility criteria. Deviation 3, appraisal instruments: AMSTAR-2 was registered but not used, as no systematic reviews were eligible. Deviation 4, reporting bias: the protocol proposed assessing it with AGREE II, AGREE-REX, AMSTAR-2 and GRADE; no formal assessment was performed, as the review synthesises recommendations rather than effect estimates. Deviation 5, outcomes: treatment, functional and quality-of-life outcomes with effect measures were registered but not analysed, for the same reason. Deviation 6, publication language: results were registered to be published in Spanish and are reported in English. Deviation 7: the review title was revised. Deviation 8, prioritisation: the registered record specified that the evidence synthesis would give precedence to recommendations from clinical practice guidelines of high methodological quality applying GRADE. One departure was applied. Fine et al. (16), the multidisciplinary collaborative consensus guidance statement on cognitive symptoms in PASC, was prioritised for the evidence synthesis although it scored 62.5% in AGREE II Domain 3, Rigor of Development, below the 70% threshold, and although its thematic scope is restricted to a single clinical manifestation. It was retained because no document meeting both criteria addressed the assessment and management of cognitive symptoms, a domain otherwise represented only by general screening statements. The exception is described in full in the Methods (Documented exceptions), the document is identified as the sole source of the recommendations concerned throughout the synthesis, and its lower Rigor of Development score is taken into account in the Limitations. Deviation 9, superseded versions: the registered record specified that previous versions of a guideline or scientific statement were not eligible. Both the preliminary guideline of the Korean Society of Infectious Diseases (17) and its most recent update (18) were appraised, and the earlier version was carried forward to the prioritised set because the update scored below the quality threshold in Rigor of Development (68.8%) and the lowest score in the corpus in Applicability (0.0%). Recommendations from Seo et al. (18) were used as complementary evidence. This decision departs from the eligibility rule as registered and is reported here rather than as routine application of the eligibility criteria.
