# Supplementary material for: Methodological quality and clinical applicability of long COVID guidelines and scientific statements: a systematic review using AGREE II and AGREE-REX
Source: Front Public Health. 2026 Sep 29;14:1930005. doi: 10.3389/fpubh.2026.1930005 (PMC13658429; doi:10.3389/fpubh.2026.1930005)
Supplement: Supplementary file 1 [file Table_1.docx]

| **Database** | **Search strategy** | **Date** |
| --- | --- | --- |
| PubMed | Search: ((Covid OR Covid19 OR "corona virus 2019" OR "coronavirus 2019" OR SARS-CoV-2 OR "B.1.1.7" OR "B.1.351" OR "B.1.1.28" OR "B.1.617" OR "BA.1" OR "BA.2" OR "BA.3" OR "BA.4" OR "BA.5" OR omicron OR deltacron OR "delta variant" OR "delta subvariant" OR "XBB.1.3") AND (PASC OR sequela* OR "post acute" OR postacute OR prolonged OR "long haul*" OR chronic OR lingering OR ongoing OR persistent OR "long term" or "more than 12 weeks" or "more than 24 weeks" OR syndrome or persist* OR lingering OR chronic OR ongoing OR long-term OR "long term" OR long-haul OR "long haul" OR convalescen* OR rehabilitat*)) OR ((Covid OR Covid19 OR "corona virus 2019" OR "coronavirus 2019" OR SARS-CoV-2 OR "B.1.1.7" OR "B.1.351" OR "B.1.1.28" OR "B.1.617" OR "BA.1" OR "BA.2" OR "BA.3" OR "BA.4" OR "BA.5" OR omicron OR deltacron OR "delta variant" OR "delta subvariant" OR "XBB.1.3") AND (PASC OR sequela* OR "post acute" OR postacute OR prolonged OR "long haul*" OR chronic OR lingering OR ongoing OR persistent OR "long term" or "more than 12 weeks" or "more than 24 weeks" OR syndrome or persist* OR lingering OR chronic OR ongoing OR long-term OR "long term" OR long-haul OR "long haul" OR convalescen* OR rehabilitat*)) AND ("Guideline"[pt] OR "practice guideline"[pt] OR "consensus development conference"[pt] OR "consensus development conference, NIH"[pt] OR guideline*[ti] OR standards[ti] OR consensus*[ti] OR recommendat*[ti] OR guideline*[cn] OR standards[cn] OR consensus*[cn] OR recommendat*[cn] OR "practice parameter*"[ti] OR "position statement*"[ti] OR "practice bulletin*"[ti] OR "policy statement*"[ti] OR CPG[ti] OR CPGs[ti] OR "best practice*"[ti] OR (care[ti] AND (path[ti] OR paths[ti] OR pathway[ti] OR pathways[ti] OR map[ti] OR maps[ti] OR plan[ti] OR plans[ti] OR standard[ti])) OR ((critical[ti] OR clinical[ti] OR practice[ti]) AND (path[ti] OR paths[ti] OR pathway[ti] OR pathways[ti] OR protocol*[ti])) OR (algorithm*[ti] AND (pharmacotherap*[ti] OR chemotherap*[ti] OR chemotreatment*[ti] OR therap*[ti] OR treatment*[ti] OR intervention*[ti])) OR (algorithm*[ti] AND (screening[ti] OR examination[ti] OR test[ti] OR tested[ti] OR testing[ti] OR assessment*[ti] OR diagnosis[ti] OR diagnoses[ti] OR diagnosed[ti] OR diagnosing[ti])) OR guideline*[ot] OR standards[ot] OR consensus*[ot] OR recommendat*[ot] OR "practice parameter*"[ot] OR "position statement*"[ot] OR "practice bulletin*"[ot] OR "policy statement*"[ot] OR CPG[ot] OR CPGs[ot] OR "best practice*"[ot] OR (care[ot] AND (path[ot] OR paths[ot] OR pathway[ot] OR pathways[ot] OR map[ot] OR maps[ot] OR plan[ot] OR plans[ot] OR standard[ot])) OR ((critical[ot] OR clinical[ot] OR practice[ot]) AND (path[ot] OR paths[ot] OR pathway[ot] OR pathways[ot] OR protocol*[ot])) OR (algorithm*[ot] AND (pharmacotherap*[ot] OR chemotherap*[ot] OR chemotreatment*[ot] OR therap*[ot] OR treatment*[ot] OR intervention*[ot])) OR (algorithm*[ot] AND (screening[ot] OR examination[ot] OR test[ot] OR tested[ot] OR testing[ot] OR assessment*[ot] OR diagnosis[ot] OR diagnoses[ot] OR diagnosed[ot] OR diagnosing[ot])) OR (("Systematic review"[ti] OR "systematic review"[pt] OR "systematic review"[ot]) AND ("practice guideline*"[tiab] OR "treatment guideline*"[tiab] OR "clinical guideline*"[tiab] OR "guideline recommendation*"[tiab])))) Filters: from 2020 - 2026 Sort by: Most Recent | 27^th^ February 2026 |
| Scopus | ( TITLE ( "practice guideline" OR "consensus development conference" OR "consensus development conference, NIH" OR guideline* OR standard* OR consensus* OR recommendat* OR "practice parameter*" OR "position statement*" OR "practice bulletin*" OR "policy statement*" OR CPG OR CPGs OR "best practice*" OR ( care AND ( path OR paths OR pathway OR pathways OR map OR maps OR plan OR plans OR standard* ) ) OR ( ( critical OR clinical OR practice ) AND ( path OR paths OR pathway OR pathways OR protocol* ) ) OR ( algorithm* AND ( pharmacotherap* OR chemotherap* OR chemotreatment* OR therap* OR treatment* OR intervention* ) ) OR ( algorithm* AND ( screening OR examination OR test OR tested OR testing OR assessment* OR diagnos* ) ) OR ( "systematic review" AND ( "practice guideline*" OR "treatment guideline*" OR "clinical guideline*" OR "guideline recommendation*" ) ) ) ) AND ( TITLE-ABS-KEY ( ( Covid OR Covid19 OR "corona virus 2019" OR "coronavirus 2019" OR "SARS-CoV-2" OR "B.1.1.7" OR "B.1.351" OR "B.1.1.28" OR "B.1.617" OR "BA.1" OR "BA.2" OR "BA.3" OR "BA.4" OR "BA.5" OR omicron OR deltacron OR "delta variant" OR "delta subvariant" OR "XBB.1.3" ) AND ( PASC OR sequela* OR "post acute" OR postacute OR prolonged OR "long haul*" OR long-haul OR "long haul" OR chronic OR lingering OR ongoing OR persist* OR "long term" OR long-term OR "more than 12 weeks" OR "more than 24 weeks" OR syndrome OR convalescen* OR rehabilitat* ) ) ) AND PUBYEAR > 2019 AND PUBYEAR < 2027 AND ( LIMIT-TO ( DOCTYPE , "re" ) OR LIMIT-TO ( DOCTYPE , "ar" ) ) AND ( LIMIT-TO ( LANGUAGE , "Spanish" ) OR LIMIT-TO ( LANGUAGE , "English" ) ) | 5^th^ March 2026 |
| Trip | Long COVID, post acute covid 19 syndrome | 7^th^ April 2026 |
| Guidelines International Network (GIN) | Long COVID, post acute covid 19 syndrome | 7^th^ April 2026 |
| National Institute for Health and Care Excellence (NICE) | Long COVID, post acute covid 19 syndrome | 7^th^ April 2026 |
| Scottish Intercollegiate Guidelines Network  (SIGN) | Long COVID, post acute covid 19 syndrome | 7^th^ April 2026 |
| World Health Organization (WHO) | Long COVID, post acute covid 19 syndrome | 7^th^ April 2026 |
| American College Of Physicians  (ACP) | Long COVID, post acute covid 19 syndrome | 7^th^ April 2026 |
